# The association of smartphone screen time with sleep problems among adolescents and young adults: cross-sectional findings from India

**DOI:** 10.1186/s12889-022-14076-x

**Published:** 2022-09-05

**Authors:** Chanda Maurya, T. Muhammad, Priya Maurya, Preeti Dhillon

**Affiliations:** 1 Department of Survey Reseach and Data Analytics, International Institute for Population Sciences, Mumbai, India; 2 International Institute for Population Sciences, Mumbai, India

**Keywords:** Screen time, Sleep problem, Adolescents, India

## Abstract

**Background:**

Although sleep problem is a growing public health issue with the advancement of technology, especially among adolescents and young adults, it received little attention. The study aimed to examine the association of screen time on smartphone with sleep problems among adolescents and young-adults in India.

**Methods:**

We used data from the “Understanding the lives of adolescents and young-adults” (UDAYA, 2018). The effective sample size for the study was 16,292 adolescents and young adults (males-4428 and females-11,864). Descriptive statistics and bivariate analysis with percentages and chi-square test were used to report the preliminary results. Multivariable logistic regression analysis was conducted to examine the association between smartphone screen time and sleep problems, separately for adolescents and young adults.

**Results:**

Nearly 15.6% of males and 23.5% of females had sleep problems in their adolescence in the last 15 days, while these percentage were high among young-adults (18.4% males and 33.24% females). Adolescents [AOR: 1.55; CI: 1.21-1.99] and young adults [AOR: 1.48; CI: 1.24-1.75], who spent more than 2 h on smartphone had higher odds of reporting sleep problems than those who did not use smartphone in the last 24 hours. Adolescent females who used smartphone for less or equal to 2 h and three or more hours respectively, had 2.11 [AOR: 2.11; CI: 1.63-2.73] and 2.94 times [AOR: 2.94; CI: 1.97-4.38] higher odds of reporting sleep problems than adolescent males who did not use smartphones. Additionally, among the young adult females, the odds of sleep problems were 1.66 times [AOR: 1.66; CI: 1.55-2.38] and 2.36 times [AOR: 2.36; CI: 1.59-3.51] greater than the non-users young adult males.

**Conclusion:**

The increased time spent on mobile phones’s screen among adolescents and young-adults, particularly in females is associated with a higher likelihood of reporting sleeping problems. The current findings have important implications for adolescence and young-adults’ mental health programmes. The findings can also be used to further inform how different strategies need to be developed for better sleep outcome during adolescence and young-adults.

## Background

Adolescence is a unique transitional phase between dependent childhood to independent adulthood, which delineates the foundation of good health of an individual’s life. Adolescents also experience rapid physical, cognitive and psychological growth [1]. Sleep is a physiological phenomenon as well as behavioural process that affects the growth, cognitive development, learning and good health of children and adolescents [2, 3]. According to the US National Sleep Foundation, the required sleep for adolescents (aged 14-17 years) is 8-10 hours and for young adults (aged 18-25 years) is around 7 to 9 hours per night to promote basic optimal health and development [4]. Unfortunately, it is an easily compromised part of daily routine. Insufficient sleep or disturbance in sleep has become a common problem among youth and adolescents worldwide [5]. Poor sleep has multiple effects on adolescent health, including depression, excessive daytime sleepiness, and metabolic dysfunctions [2, 3, 6]. Previous evidence identified various social, environmental, cultural and family-related factors responsible for sleep disorders among adolescents and young adults [5, 7–9].

Since the beginning of the new millennium, the countries around the world have witnessed an era of continued technological advancement, resulting in an increase in people’s screen viewing of digital devices such as television, mobile phones, other portable electronic devices, and the internet [10, 11]. Although the internet penetration rate, which is the percentage of total population of a country or region using internet, is high in the developed world (86.6%) than developing world (47%) and least developed countries (19.1%), a rapid increase has been observed in low and middle-income countries in recent years [11]. A recent survey in India reported that 92.8% of the households had mobile ownership [12], and 35% of the country’s total population had internet access in 2017 [11]. Particularly, more than two-third (67%) of internet users belonged to the age group of 12 to 29 years and about one-third (32%) of them were from 12 to 19 age cohort in the country [13, 14]. Increased availability of smartphones and accessibility of internet come up with access and use of the internet during daytime and bedtime, further which leads to internet addiction [15–17]. In 2016, the new Canadian 24-hour Movement Guidelines recommended that screen time/day should be less than 2 h for children and adolescents [18].

The use of smartphones has a wide range of positive impacts, such as updated information and improved academic performance; however, the negative impacts include substance abuse and addiction to the device affecting individuals’ social and personal life [8, 15, 17, 19]. One of the major negative impacts is the sleep disturbance that leads to other problems among adolescents and young adults. Problematic use of smartphones has been attributed to time displacement, such as smartphone use that transcends to delay in bedtime due to surfing the content of the media, causing arousal and interfering with the ability to fall asleep. Additionally, it has a biological impact through light emission of the device in the blue spectrum resulting in melatonin suppression which further leads to difficulty in sleep initiation and non-restorative sleep [6–8, 16, 17, 19]. With the advancement of technology and the use of smartphones, the sleep problem has become a growing concern. Age, gender, physical activities and substance use are other potential risk factors for sleep disorder among adolescents [20–22]. A systematic review revealed that adolescents sleep less as they get older. The same study also mentioned that females sleep more than males but females’ bed time is decreasing at a larger rate than males for each year of increasing age [20]. Heavy drinking behaviour,smoking and physical activities are significantly associated with sleep disorders among adolescents [21].

Although sleep problem is a growing public health issue with the advancement of technology, especially among adolescents and young adults, it received little attention in India. Study reported that magnitude of smartphone addiction among teens and youngsters in India ranged from 39 to 44% [23]. Similarly, sleep disorders are usually prevalent among adolescents and young adults due to lifestyle factors, dietary habits and hormonal and emotional disturbances [24]. However, sleep problem independently associated with smartphone screentime is a neglected topic among youngsters in India. This study can help to understand the linkage between screen time and sleep problems during adolescence and early adulthood. Also, itmay suggests policy implications that can help Indian adolescents and youth improve their mental health and achieve better academic performance through limited screen time and good sleep behaviour. Thus, the present study aimed to examine the association of smartphone screen time with sleep problems among adolescents and young adults in India after controlling for a large number of confounders.

## Materials and methods

### Data

The data for this study were derived from the survey of “Understanding the lives of adolescents and young adults” (UDAYA, 2018), conducted by the Population Council, New Delhi and funded by the Bill and Melinda Gates Foundation and the David and Lucile Packard Foundation. The UDAYA survey is a longitudinal study conducted in Uttar Pradesh and Bihar following a cohort of adolescents aged 10-19 years.

The UDAYA study used both cross-sectional and longitudinal designs for sampling at wave- 1, and a multi-stage systematic sampling design was employed during the sample selection. The UDAYA was designed to provide estimates at two time-points for the state as well as for the urban and rural areas of the state for each of the five categories of respondents, namely younger males in the age group 10–14 years, older males in the age group 15–19 years, younger females in the ages group 10–14 years, unmarried older females in the age group 15–19 years, and married older females in the age group 15–19 years. A total of 150 primary sampling units (PSUs), 75 for rural and 75 for urban respondents, were sampled in each state using the probability proportional to size (PPS) technique. PSUs’ list was stratified using four variables, namely, region, village/ward size, the proportion of the population belonging to scheduled castes and scheduled tribes, and female literacy. The household sample in rural areas was selected in three stages, while in urban areas, it was selected in four stages.

Data collection for wave-1 was done during 2015-16, and after 3 years, wave-2 data were collected during 2018-19. This paper analysed smartphone screen time in the past 24 hours, and this information was collected only in wave-2. Hence, for the current study, a cross-sectional sample of only wave-2 was used, consisting of 12- 23 years old adolescents and young adults. The effective sample size for the study was 16,292 adolescents and young adults (males-4428 and females-11,864).

### Variable description

#### Outcome variable

The sleep problem was coded as 1 “having sleep problems in the last 15 days” and 0 “not having sleep problem in the last 15 days”.

#### Explanatory variables

Key explanatory variable was time spent on smartphone in the past 24 hours that was coded as 0 “not users of smartphone in past 24 hours”, 1 “one to two hour of smarphone use” and 3 “three or more hours of smartphone use” [25]. Socio-demographic variables included age, that was grouped into “12-18 years” and “19-23 years”; current marital status, that was coded as “single” and “married”; educational level, that was coded as 0 “illiterate”, 1 “primary and middle for up to 8 years of schooling” and 3 “higher for nine and more years of schooling”. Other predictor variables were social media exposure, that was coded as 0 “no exposure” and 1 “exposure”; physical activity, that was coded as 0 “no physical activity in the past week”, low activity “less than 7 hours of physical activity in the past week” and high activity “7 or more hours of physical activity in the past week” [26]; sedentary behaviour, that was coded as “no sedentary behaviour in the past week”, “less than or equal to 14 hours of sedentary behaviour in the past week”, and “more than 14 hours of sedentary behaviour” [26]; substance use, that included consumption of either tobacco products or alcohol, which was coded as “no” and “yes”; parent’s co-residence with the respondents, that was coded as “both parents co-reside”, “one parent co-resides”, and “no parents co-reside”; paid work in the past 12 months, that was coded as “no” and “yes”. The control variables also included religion, that was coded as “Hindu” and “non-Hindu”; caste, that was coded as “Scheduled Caste/Scheduled Tribe (SC/ST)”, “Other background class (OBC)”, and “other”; mother’s education, that was coded as “no” and “yes”; the wealth index, that was coded as “poor”, “middle”, and “rich”; and the place of residence, that was coded as “rural” and “urban”.

### Statistical analysis

Descriptive statistics and bivariate analysis with percentages and chi-square test were used to report the preliminary results. Two multivariable logistic regression models were used to analyse the association between the binary outcome variable and explanatory variables, separately for adolescents and young adults. The outcome variable was (0 “not sleep problem” and 1 “having sleep problem”). To assess the reliability of the models we calculated the Hosmer-Lemeshow goodness-of-fit statistic [27]. Both regression models showed good calibration (Hosmer-Lemeshow *P* value of 0.52 and 0.59, respectively). The results were presented in the form of the adjusted odds ratio (OR) with 95% confidence interval (CI) and interaction term was used to identify the interaction effects of predictors of sex and time spent on smartphones on sleep problem. The logistic regression model is usually put into a more compact form as follows:

$$\mathrm{In}\left(\frac{{\mathrm{p}}_{\mathrm{i}}}{1-{\mathrm{p}}_{\mathrm{i}}}\right)={\upbeta}_0+{\upbeta}_1{\mathrm{x}}_1+\dots +{\upbeta}_{\mathrm{M}}{\mathrm{x}}_{\mathrm{m}-1},$$

Where β0… βM are regression coefficients indicating the relative effect of a particular explanatory variable on the outcome. These coefficients change as per the context in the analysis in the study.

## Results

Socio-demographic profile of adolescent and young adult males and females are presented in Table 1. A higher proportion of adolescent males (98.68%) and females (82.02%) were single, while these percentage were low among young adult males (88.62%) and females (37.27%). A proportion of 48.21 and 78.69% of the adolescents and young adult males and 52.11 and 56.17% of the adolescent and young adult females, respectively had high school and above educational level. Fourteen percent of males and only 4 % of females in their adolescence spent three or more hours on smartphone, while these percentages were higher among young adults (25.59% males and 5.79% females). Nearly 58 and 53% of adolescent males and females reported smartphone screentime of less than 2 h. There were significant sex differences in the exposure to social media among both adolescents and young adults. One-fifth of the adolescent males and a quarter of the adolescent females were exposed to the social media, while 44% of the males and 5% of the females were exposed to social media in their young adulthood. Approximately 15.64 and 23.52% of adolescent males and females respectively had sleep problems in the last 15 days of the interview, while these percentages were higher among young adults (18.4% males and 33.24% females).

**Table 1:** Socio-demographic characteristics of the study sample

Background variable	Adolescents		Young adults	
	Male	Female	Male	Female
**Time spent on mobile phone**				
None	28.44	39.94	9.00	28.41
= < 2 hour	57.66	56.30	65.41	65.80
> 2 hour	13.90	3.76	25.59	5.79
**Marital status**				
Married	1.32	17.98	11.38	62.73
Single	98.68	82.02	88.62	37.27
**Education**				
Illiterate	1.73	6.31	3.06	14.44
Primary & middle	50.06	41.57	18.25	29.39
High school & inter & above	48.21	52.11	78.69	56.17
**Social media exposure**				
No	79.67	96.41	56.08	95.23
Yes	20.33	3.59	43.92	4.77
**Physical activity**				
No	60.84	82.83	49.79	85.89
Low activity	21.26	10.59	21.88	8.26
High activity	17.9	6.58	28.33	5.85
**Sedentary behaviour**				
No	25.72	31.14	24.82	34.18
< =14 hours	63.32	57.53	61.66	54.65
> 14 hours	10.96	11.33	13.52	11.18
**Sleep problem**				
No	84.36	76.48	81.6	66.76
Yes	15.64	23.52	18.4	33.24
**Substance use**				
No	79.67	96.41	56.08	95.23
Yes	20.33	3.59	43.92	4.77
**Number of friends**				
No	1.43	4.7	1.71	10.83
1-4	49.33	58.57	43.84	61.37
More than 4	49.24	36.73	54.45	27.8
**Parental coresidence**				
No	1.91	2.02	3.05	1.48
Only one parent	14.23	14.15	14.66	7.91
Both	83.85	83.84	82.29	90.62
**Paid work in last 12 months**				
Yes	29.3	22.17	62.84	21.87
No	70.7	77.83	37.16	78.13
**Religion**				
Hindu	84.93	80.34	84.94	78.56
Non-Hindu	15.07	19.66	15.06	21.44
**Caste**				
SC/ST	25.77	26.29	29.19	26.09
OBC	57.56	56.26	51.4	54.77
Others	16.67	17.45	19.41	19.13
**Mother’s education**				
Illiterate	70.53	72.31	69.05	75.61
Literate	29.47	27.69	30.95	24.39
**Wealth Index**				
Poor	35.39	35.81	26.46	29.63
Middle	21.75	22.27	22.86	21.58
Rich	42.86	41.92	50.68	48.79
**Place of residence**				
Urban	15.67	15.1	18.1	15.71
Rural	84.33	84.9	81.9	84.29
**State**				
Uttar Pradesh	63.53	61.03	73.15	72.71
Bihar	36.47	38.97	26.85	27.29
**Total(N)**	**2399**	**3432**	**2029**	**8432**

**Fig. 1:**
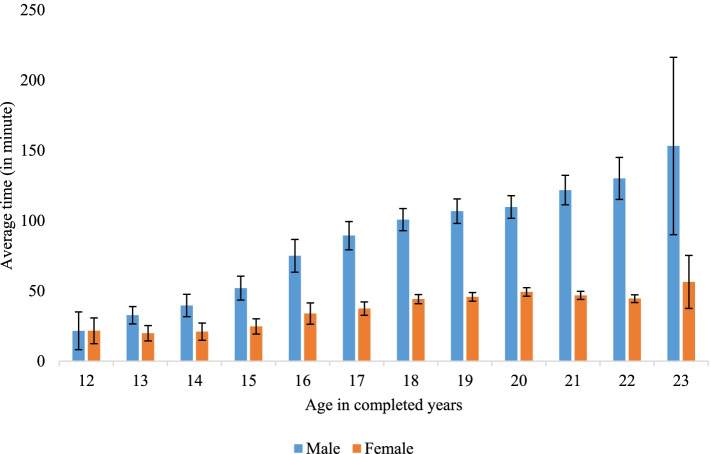
Average screentime spent on smartphone among adolescents and young-adults, stratified by sex

Figure 1 depicts the average time (in minutes) spent on smartphones increases with age, irrespective of sex of the respondents. Adolescent males spent more time on smartphones than adolescent females, and the sex difference in smartphone screentime increased with age. For instance, at age 23 years, adolescent males spent around 3 hours (153 minutes), and adolescent females spent an hour (56 minutes) on smartphones in the last 24 hours preceding the survey.

**Fig. 2:**
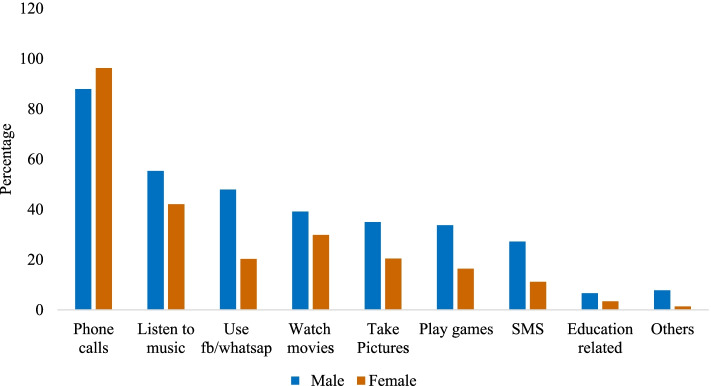
Things adolescents and young adults did on smartphone, stratified by sex

Figure 2 depicts the type of activities adolescents and young adults did on a smartphone. Only 7% of males and 3% of females used their smartphones for educational purposes, whereas, a higher percentage of males and females used their smartphones for phone calls. Half of the males and one out of five females used their smartphones for surfing social media.

The association of sleep problem among adolescents and young adults with background characteristics are shown in Table 2. The results in the table showed clear sex differentials for sleep problems among adolescents and young adults. Result revealed that a higher percentage of adolescents who used smartphone for three or more hours suffered from sleep problem in last 15 days (Males: 22.46% and females: 38.51%) and a higher percentage of young adults who used smartphone for three or more hours had sleep problem (males: 23.47% and females: 44.34%). It was revealed that both adolescents and younger adult females who used smartphone for three or more hours had higher percentage of sleep problem in comparison their male counterpartsmale.

**Table 2:** Bivariate association of sleep problem and background characteristics among adolescents and young adults, stratified by sex

Variables	Adolescents		Female-Male difference	Young adults		Female-Male difference
	Female	Male	***p*** < 0.05	Female	Male	***p*** < 0.05
**Time spent on mobile phone**						
None	20.86	14.61	6.25*	31.32	26.74	4.58*
= < 2 hour	30.23	16.26	13.97*	37.32	19.51	17.81*
> 2 hour	38.51	22.46	16.05*	44.34	23.47	20.86*
**Marrital status**						
Unmarried	24.76	16.88	7.88*	28.57	21.49	7.08*
Married	37.98	10.34	27.63*	41.19	19.77	21.42*
**Education**						
Illiterate	29.75	19.15	10.60*	39.23	29.69	9.54*
Primary and secondary	25.08	13.92	11.15*	37.27	18.32	18.96*
Higher	28.53	19.62	8.90*	35.03	21.63	13.40*
**Social media exposure**						
No	25.25	12.38	12.87*	35.60	18.87	16.73*
Yes	32.40	19.79	12.61*	37.88	21.99	15.89*
**Physical activity**						
No	25.93	15.57	10.36*	34.56	18.86	15.71*
Low activity	36.83	20.33	16.50*	48.92	29.04	19.88*
High activity	29.61	17.10	12.51*	42.95	20.15	22.80*
**Sedentary behaviour**						
No	28.44	16.54	11.90*	35.49	18.98	16.51*
< =14 hours	26.38	15.66	10.73*	37.05	21.27	15.78*
> 14 hours	28.99	23.10	5.88*	34.81	25.00	9.81*
**Substance use**						
No	26.93	16.41	10.52*	36.02	19.79	16.23*
Yes	36.67	18.33	18.33*	42.73	23.39	19.34*
**Number of friends**						
No	25.44	11.76	13.68	35.15	6.25	28.90*
1 to 4	26.02	13.86	12.16*	36.01	19.75	16.26*
More than	29.37	19.11	10.25*	37.30	22.83	14.47*
**Parental coresidence**						
No	27.59	15.38	12.20	24.52	22.37	2.15
Only one parent	27.01	15.90	11.11*	34.26	25.08	9.18*
Both	27.31	16.99	10.32*	36.72	26.33	10.40*
**Paid work in last 12 months**						
No	26.87	0.16	26.71*	35.88	19.34	16.54*
Yes	28.89	19.40	9.48*	37.77	22.70	15.07*
**Religion**						
Hindu	27.59	16.63	10.96*	36.52	21.80	14.72*
Non-Hindu	26.18	17.74	8.44*	35.44	18.96	16.48*
**Caste**						
SC/ST	29.38	15.51	13.88*	37.98	21.88	16.10*
OBC	27.24	17.41	9.83*	36.45	21.17	15.28*
Others	24.60	16.55	8.05*	33.36	21.14	12.21*
**Mother’s education**						
Illiterate	27.96	16.28	11.68*	36.98	21.84	15.15*
Literate	25.70	17.80	7.89*	34.38	20.46	13.92*
**Wealth index**						
Poor	28.60	15.43	13.17*	38.54	19.13	19.41*
Middle	27.61	13.43	14.18*	37.81	18.83	18.98*
Rich	26.31	19.00	7.31*	34.44	22.95	11.49*
**Place of residence**						
Urban	30.68	19.07	11.61*	36.95	23.93	13.02*
Rural	24.72	15.16	9.56*	35.83	18.91	16.92*
**State**						
Uttar Pradesh	20.43	12.77	7.67*	30.13	17.33	12.79*
Bihar	32.72	20.67	12.05*	41.86	26.33	15.53*

Moreover, a higher percentage of females (29.75 39.23%) who never attended school suffered from sleep in their adolescence and young adulthood, respectivily. There were higher and significant sex differences in sleep problems (13.40%) among the young adults who had higher educational attainments. Significant gender diffecences found in sleep problems among young adults who were exposed to social media. The prevalence of sleep problems was higher among adolescent and young adult males (20.3 and 29%) and females (36.8 and 48.9%) with low physical activity.

Furthermore, adolescents (males: 18.3% and females: 36.7%) and young adults (males:23.4% and females:42.7%) who used substances had a significantly higher prevalence of sleep problems. Adolescent and young adult males and females who had one to four or more than four friends had a significantly higher prevalence of sleep problems than those who did not have friends. The adolescents (males: 19.1% and females: 30.7%) and young adults (males: 23.4%% and females: 36.9%) who belonged to an urban residence had a substantially higher prevalence of sleep problems than those who belonged to a rural place.

Estimates from logistic regression analysis of sleep problems across explanatory variables among adolescent and young adult males and females are presented in Table 3. Adolescents and young adults who used smartphones for more than 2 h had 1.55 times [AOR: 1.55; CI: 1.21-1.99] and 1.48 times [AOR: 1.48; CI: 1.24-1.75] higher odds of suffering from sleep problems in comparisonto those who did not use smartphone in last 24 hours. Moreover, there was a considerable sex differentce in reporting sleep problem. The odds of sleep problems were 2.08 times [AOR: 2.08; CI: 1.77-2.44] and 2.55 times [AOR: 2.55; CI: 2.17-2.99] higher among adolescent and young adult females compared to their male counterparts.

**Table 3:** Estimates from logistic regression on reporting of sleep problems among adolescents and young adults by their background characteristics

Background characteristics	Adolescents	Young adults
	AOR(95% CI)	AOR(95% CI)
**Time spent on mobile phone**		
None®		
= < 2 hour	1.23***(1.05 1.43)	1.12**(1.01 1.25)
> 2 hour	1.55***(1.21 1.99)	1.48***(1.24 1.75)
**Sex**		
Male®		
Female	2.08***(1.77 2.44)	2.55***(2.17 2.99)
**Marital status**		
Single®		
Married	1.66***(1.36 2.02)	1.7***(1.53 1.89)
**Education**		
Never®		
Primary & middle	0.95 (0.7 1.27)	1.01 (0.88 1.17)
High school & inter & above	1.17 (0.87 1.58)	1.04 (0.91 1.2)
**Social media exposure**		
No®		
Yes	1.25***(1.07 1.46)	1.1*(0.98 1.22)
**Physical activity**		
No®		
Low activity	1.5***(1.25 1.79)	1.85***(1.62 2.11)
High activity	1.16 (0.93 1.43)	1.36***(1.17 1.59)
**Sedentary behaviour**		
No®		
< =14 hr	0.8***(0.69 0.94)	1.02 (0.92 1.13)
> 14	0.9 (0.72 1.13)	0.97 (0.83 1.12)
**Substance use**		
No®		
Yes	1.05 (0.84 1.32)	1.24***(1.06 1.46)
**Number of friends**		
No®		
1 to 4	1.15 (0.81 1.64)	1.16*(1 1.34)
More than 4	1.37*(0.96 1.97)	1.28***(1.09 1.5)
**Parental coresidence**		
No		
Only one parent	0.88 (0.59 1.34)	0.73**(0.53 1)
Both®	0.94 (0.78 1.13)	1.1 (0.95 1.27)
**Paid work in last 12 months**		
No®		
Yes	1.17*(1 1.36)	1.22***(1.1 1.35)
**Religion**		
Hindu	0.93 (0.78 1.11)	0.92 (0.82 1.03)
Non-Hindu®		
**Caste**		
SC/ST	1.18 (0.95 1.47)	1.05 (0.9 1.21)
OBC	1.06 (0.88 1.27)	0.96 (0.85 1.09)
Other®		
**Mother’s education**		
Illiterate®		
Literate	0.9 (0.77 1.05)	0.94 (0.84 1.04)
**Wealth Index**		
Poor	1.05 (0.88 1.26)	1.05 (0.93 1.18)
Middle	0.97 (0.81 1.16)	1.04 (0.93 1.17)
Rich®		
**Place of residence**		
Urban®		
Rural	0.75***(0.65 0.87)	0.89**(0.81 0.98)
**State**		
Uttar Pradesh®		
Bihar	1.67***(1.46 1.91)	1.53***(1.4 1.67)
Constant	0.09***(0.06 0.16)	0.09***(0.07 0.12)

*AOR* Adjusted odds ratio *CI* Confidence Interval,® Reference category, *SC/ST* Scheduled Caste/Scheduled Tribe, *OBC* Other backword class

^*^p < 0.1

^**^p < 0.05

^***^p < 0.01

Estimates from logistic regression on reporting sleep problems among adolescents and young adults by interaction between sex and time spent on smartphone are presented in Table 4. The interaction analysis found that the odds of sleep problems among young adult males who used smartphones for 1 or 2 h were lower [AOR: 0.63; CI: 0.44-0.92] than non-users from young adult males. Adolescent females who used smartphone for less or equal to 2 h and three or more hours respectively, had 2.11 [AOR: 2.11; CI: 1.63-2.73] and 2.94 times [AOR: 2.94; CI: 1.97-4.38] higher odds of reporting sleep problems than adolescent males who did not use smartphones. Among the young adult females, the odds of sleep problem were 1.66 times [AOR: 1.66; CI: 1.55-2.38] and 2.36 times [AOR: 2.36; CI: 1.59-3.51] greater than the non-users from young adult males.

**Table 4:** Estimates from logistic regression on reporting of sleep problems among adolescents and young adults by interaction between sex and time spent on mobile phone

Background characteristics	Adolescents	Young adults
	AOR(95% CI)	AOR(95% CI)
**Sex # Time spent on mobile phone**		
Male # No time spent on mobile phone®		
Male # Spent 1 to 2 hour on mobile phone	0.92 (0.7 1.21)	0.63**(0.44 0.92)
Male # Spent 3 or more hour on mobile phone	1.13 (0.8 1.61)	0.77 (0.51 1.14)
Female # No time spent on mobile phone	1.54***(1.17 2.03)	1.42*(0.98 2.06)
Female # Spent 1 to 2 hour on mobile phone	2.11***(1.63 2.73)	1.66***(1.15 2.38)
Female # Spent 3 or more hour on mobile phone	2.94***(1.97 4.38)	2.36***(1.59 3.51)
**Constant**	0.12***(0.07 0.2)	0.16***(0.11 0.25)

Interaction analyses were performed after controlling for selected background characteristics

*AOR* Adjusted odds ratio, *CI* Confidence Interval,*®* Reference category

^*^p < 0.1

^**^p < 0.05

^***^p < 0.01

## Discussion

The aim of this study was to examine the prevalence of screen time and sleep problems and associations of time spent per day on screen, and the reporting of sleep problems in adolescents and young adults in India. A proportion of 13.90 and 25.59% of adolescent and young males and 3.76 and 5.79% of adolescent and young females reported increased time spent on mobile phones per day, which was higher than the recommended total screen time to minimise the negative health effects in previous studies [7, 28]. Screen time is negatively associated with markers of health in adolescents and young adults in developed countries [9, 29, 30], but very little is known about such relationships in these populations in low- and middle-income countries. Studies in China found that increased screen time is associated with adolescents’ unhealthy behaviours and undesirable psychological states that can contribute to sleep problems and poor quality of life [31–33]. Consistently, the current findings showed that adolescents and young adults who reported increased screen time had higher odds of sleep problems, and greater odds were observed among adolescents than young adults. Another study in the US showed that screen time on mass media such as reading news online and social media was associated with increased odds of short sleep duration [34]. Similarly, the likelihood to have insufficient sleep was higher for adolescents who engaged in excessive screentime behaviours when compared to those who did not engage in such behaviours [35].

Given the number of studies demonstrating the adverse effects of insufficient sleep on adolescents’ and young adults’ physical and mental health, the increasing proportion of those who do not get the recommended hours of sleep raises public health concerns. On the other hand, a large body of literature has shown that adolescent and young females suffer more frequently from sleep disorders than males [36–38], and the current study has supported this suggesting a call for special attention for the development of gender-based initiatives by health-decision makers and policy experts in the country. Excessive screen exposure in some studies was shown to be associated with poor psychosocial well-being, and sleep played a mediating role [39]. Similarly, multiple processes have been identified as potential mechanisms responsible for the negative impacts of screen time on sleep, including displacement of sleep time, increases in arousal that harm sleep quality, re-entrainment of circadian rhythms due to light exposure, and increases in depressive symptoms [40–43].

Furthermore, beyond the screen time- sleep problem association, we were able to demonstrate different associations for both adolescent and young males and females by doing interaction analyses. In concordance with earlier findings [44], the current study showed that adolescent and young females who spent more time on screen had a higher likelihood of reporting sleep problems in comparison to adolescent and young males who reported no screen time. The observed gender difference may be attributed to discrepancies in their patterns of use and the nature of the content. For example, gender difference can be noticed in the motivations behind attending or listening to music; while males may consider music as a means to create a more positive image of themselves or boost their energy level, whereas females tend to listen to music as a reflection of their current negative emotional state including feeling lonely or depressed [45, 46]. Therefore, while considering the future studies on gender differences in the observed association, the types of media and content as markers of such gender difference should be analysed.

In addition to excessive screentime behaviors, the following factors were also found to be associated with reporting sleep problems: increasing age, physical activity, substance use, higher number of friends, engaged in paid work, and urban place of residence. As prior research documented that increasing age is negatively associated with sleep quality and positively associated with sleep problems [47–50], the finding of our study consistently showed that reporting of sleep problem varied by age, with early to mid adolescents (12-17 years of age) reporting lesser sleep problem than late adolescents (18–23 years of age). Physical activity in the current study was positively associated with sleep problems which is inconsistent with multiple previous studies showing that physical activity has a protective effect on insufficient sleep and related stress during adolescence [51, 52]. The inconsistent finding may partially be explained by the possible reverse causality that suggests that earlier onset of sleep predicts increased sedentary behaviour and less physical activity in the next day [53].

The cross-sectional design of the study precludes determining the causation in the observed associations. Besides, we did not separate out the types of screen time and content and sleep on school-day and non-school-day or weekend and weekday. These are important while concluding the findings; however, research has to be further conducted covering these aspects. The self-reported nature of screen time and sleep problems is subject to measurement error due to recall and social desirability bias. Further studies by including subjective and objective measures of both variables and detailed information on sleep disorder such as shortened sleep duration, longer sleep latency, and more mid-sleep awakenings need to be undertaken. A major limitation of the study is to evaluate cell phone use only in the previous 24 hours. Similarly, we evaluated sleep-related problems in the last 15 days. However, peculiar situations could modify the sleep referred to moment, since it is a short period, thus modifying the result found.

Furthermore, it is plausible that the mechanisms by which increased time spent on mobile phones is related to reporting sleep problems, which in turn is related to behavioural health, may differ depending on the adolescents’ developmental stage. For example, late adolescents may engage in more screen time and deliberately avoid sleep, whereas young adolescents may be overstimulated by the games and other online activities and, therefore, have more difficulty settling in when it is time to sleep. Future investigation is warranted on the trajectories of sleep disorders during adolescence. On the other hand, although the current results support a possible causal inference that increased screen time per day (more than 2 hours) is responsible for reporting sleep disorder using large scale survey data, there is a need for longitudinal and randomised-control intervention studies that may strengthen the causal inferences and explore specific processes responsible for this influence. The study was conducted during pre-CoVID-19 pandemic, and therefore, with the increase in online learning and reduction in outdoor activities, the screen time use might have increased among adolescence. Further, the study setting is from the lower socio economic states with prominent rural areas, screen time use in other states and urban areas of India may be much higher than that is reported in the present study. Therefore, the effect of screentime use on sleep disorders may be higher in current scenario in Indian setting than the reported in present study.

## Conclusions

The increased time spent on the mobile phone among adolescents and in females, in particular, is associated with a higher likelihood of reporting sleeping problems. The current findings have important implications for health practitioners and families with adolescent members and mental health programmes in adolecence. The findings can also be used to inform further how different strategies need to be developed for sleep health during adolescence. Future studies are required to explore the potential interventions that uniquely target adolescents who have poor sleep health.

## Data Availability

The study utilizes a secondary source of data that is freely available in the public domain through: https://dataverse.harvard.edu/dataset.xhtml?persistentId=doi:10.7910/DVN/RRXQNT. The necessary ethical approval has been taken by the respective organizations involved in the data collection process.
